# Ag Nanoparticles for Biomedical Applications—Synthesis and Characterization—A Review

**DOI:** 10.3390/ijms23105778

**Published:** 2022-05-21

**Authors:** Alexandra Nicolae-Maranciuc, Dan Chicea, Liana Maria Chicea

**Affiliations:** 1 Research Center for Complex Physical Systems, Faculty of Sciences, Lucian Blaga University of Sibiu, Dr. Ion Raţiu Street 5−7, 550012 Sibiu, Romania; alexandra.nicolae@ulbsibiu.ro; 2 Faculty of Medicine, Lucian Blaga University of Sibiu, 550169 Sibiu, Romania; liana.chicea@ulbsibiu.ro

**Keywords:** Ag, silver nanoparticles, synthesis, functionalization, size, shape, characterization techniques

## Abstract

Silver nanoparticles have been intensively studied over a long period of time because they exhibit antibacterial properties in infection treatments, wound healing, or drug delivery systems. The advantages that silver nanoparticles offer regarding the functionalization confer prolonged stability and make them suitable for biomedical applications. Apart from functionalization, silver nanoparticles exhibit various shapes and sizes depending on the conditions used through their fabrications and depending on their final purpose. This paper presents a review of silver nanoparticles with respect to synthesis procedures, including the polluting green synthesis. Currently, the most commonly used characterization techniques required for nanoparticles investigation in antibacterial treatments are described briefly, since silver nanoparticles possess differences in their structure or morphology.

## 1. Introduction

Nanotechnology, a research field that has been in constant development since 1959, represents an unique and innovative research approach [1,2]. Described first in 1974 by Norio Taniguchi and then experimentally proven in 1981 by two IBM researchers who developed the scanned tunneling microscopy [1], the field of nanotechnology has gained significant interest in the scientific domain. The big potential of this scientific domain is related to the ingress in atoms and molecule investigation. The possibility of creating machines at nanoscale dimensions and the fabrication of devices with a specific atom distribution become a priority for certain researchers or engineers. Nanotechnologies developed until now create low dimensional materials with unique properties which offer the ability to be used in many domains, such as the textile industry, medicine, electronics, or automatics. Nanotechnology can be considered an innovative field of material science due to the particular advantages offered by it. In recent years, the economy has shown improvements regarding the need for smaller and better materials in all domains. As well as its small dimensions, nanomaterials have gained attention for their physicochemical properties, such as conductivity or optic activity [3].

Nanomaterials have changed the classical investigation models. The need for light and sustainable products, targeted therapies, and optimized diagnostic devices has allowed for the discovery of the beneficial contributions of nanomaterials. In medicine, these nano-dimensions are studied more and more. Their most important property, a larger surface-volume ratio compared to the bulk size, increases their activity in biological systems [4].

**Figure 1:**
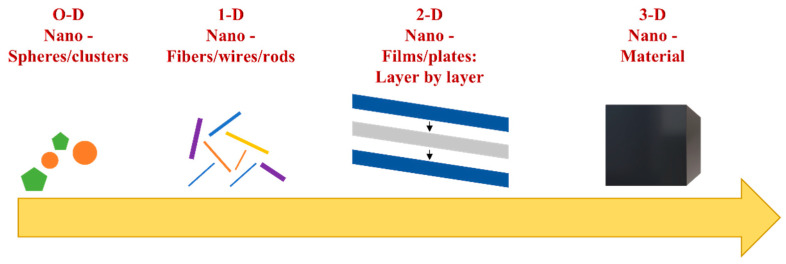
Classification of nanomaterials regarding their dimensions.

Materials at the nanoscale level are found in a variety of forms depending on their aspect and structure. Over time, many criteria were considered for describing nanomaterials. Origin, form, or dimensional characteristics have led to a variety of classification criteria for nanomaterials. In literature studies, the most important criterion is based on dimensional properties [2,5]. In biomedical applications, dimension is an essential factor because the interaction of materials with biological structures inside the human body strongly depends on it. In this regard, the nanomaterials can be classified in 0-D, 1-D, 2-D and 3-D dimensions, depending on the architecture and on the external sides of the material, as can be observed in Figure 1 [2,5,6].

## 2. Nanoparticle Types

Nanoparticles, an important field of nanotechnology, are materials with nanometric dimensions less than 100 nanometers and are considered to be 0-D materials. The specific and well-defined properties of nanoparticles offer them advantages in variable engineering applications such as medicine, chemical engineering, automotive, electronics, and even in the food industry. The form and the dimension are the main factors studied in the research community. These factors can influence the physical–chemical and optical properties of nanoparticles. The concentration of nanoparticles in solutions, the density or the color are other factors which induce modifications in nanoparticles-based materials. Each application requires certain properties, so the dependence of size or shape on the final application is recognized in the literature studies.

Nanoparticles are complex molecules with structures composed of three layers. The outside layer allows for the functionalization with other molecules, such as metal ions or polymers. The middle layer, chemically different from the core, assures the bond between the inner of the particle and the outside part. The core of the nanoparticle represents the nanoparticle’s material itself [7]. The functionalization with various active bio-substances has gained the researchers’ attention. They developed complex systems for tissue engineering. Coating some nanoparticles with polymers such as chitosan or dextran assures an antimicrobial character in the polymeric hydrogel obtained as such [8]. In addition, a recent strategy in the medical research field is based on the encapsulation of bio-substances or drugs [9] with the purpose of stimulating the regeneration of the injured organ during the trauma.

**Figure 2:**
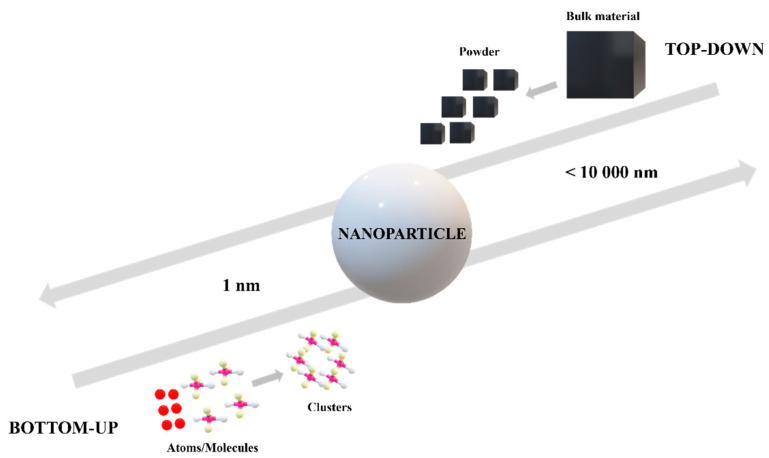
Top-down methods versus bottom-up methods from a nanoparticles perspective.

Nanoparticles can be synthesized through different approaches. In general, there are two directions: “top-down” and “bottom-up” methods. Depending on the precursors and materials used, the two approaches are based on different technologies. The “top to bottom” method is based on a bulk material transformation into small particles, while the “bottom to top” method involves the production of nanoparticles using chemical reagents able to assemble atoms to “seed” nuclei which will grow further into clusters and particles of nanometric dimensions, as can be observed in Figure 2 [10,11,12]. Organic particles are usually obtained through bottom-up methods such as chemical reduction, sol-gel, emulsification, or self-assembly processes. These methods lead, in most cases, to nanoparticle fabrication in a spherical form and in a polydisperse size distribution due to the inherent surface tension that makes itself manifest. Besides organic particles, inorganic nanoparticles in different shapes can be obtained through bottom-up techniques, as well, for example, by a nucleation process [13].

Top-down methods involve the use of physical techniques in nanoparticle synthesis. Starting from a bulk material and using electron-related techniques in most cases, nanoparticles can be obtained. Photolithography, nanoimprint lithography, mechanical processes, or electronic beam lithography are top-down methods to fabricate nanosized materials [13,14].

Apart from the two approaches, a hybrid method has been developed lately. To overcome the disadvantages that appear through the classical techniques, a combination of top-down and bottom-up methods is described as a hybrid approach [12,15].

**Scheme 1:**
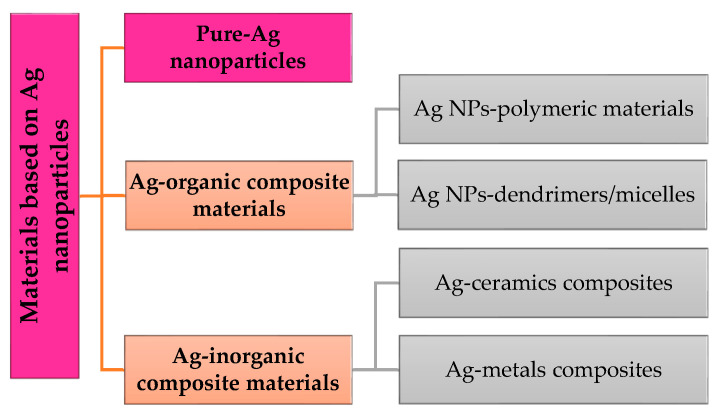
Materials based on Ag nanoparticles.

Nanoparticles can be classified through their origin and their composition. They can be found in interactions with organic particles (dendrimers, liposomes), with inorganic materials (metals in general), and with materials and particles based on carbon (graphene, nanotubes). Silver (Ag) is obtained through mechanical tries from metals, so it is classified as an inorganic material with high resistance. In this regard, a simple classification of materials based on silver nanoparticles (Ag NPs) is described in Scheme 1.

- *Ag-organic composite materials*

Organic nanoparticles are usually obtained from organic matter. Their organic nature transformed these nanoparticles into a favorite type regarding the drug release applications and tissue regeneration. The weak interactions that appeared between molecules led to the possibility of transforming these organic nanoparticles into dendrimers, micelles, or liposome systems [3] able to encapsulate a desired drug for controlled release. Dendrimers, a multi-branch structure with a 3-D form, is one of the latest approaches in drug delivery systems [16]. Able to encapsulate different molecules in their cavities, they are suitable for targeted therapies in the case of cancer or other aggressive diseases.

Ag-organic composite materials seem to combine the stability of silver with the biocompatible characteristics of organic compounds. In tissue regeneration and antibacterial applications, two of the most studied natural polymers in the biomaterials branch are chitosan and collagen, due to their synergistic effects. Able to assure a faster regeneration due to collagen, silver antibacterial potential is increased by the chitosan as well. Collagen, the most important protein from our body and the easiest polymer to use [17], is increasing the biocompatibility of Ag-based materials. Its advantages, such as water dissolution, low cost, and variety, depending on the need of applications, led to the proposal of different materials for biomedical applications: sponges with Ag embedded, films, hydrogels, or even blends. Most of the literature studies regarding collagen-based silver nanomaterials carried out the maintenance of the nanosized dimension of silver particles, while being mixed in formulations with antibacterial applications [18,19,20]. A recent study performed by Novaes et al. [21] revealed that Ag NPs can also be entrapped in blends for a long-lasting release effect. Using a mix of chitosan and collagen in different rations to form blends, the final material achieved open pores with high interconnectivity between them able to encapsulate the Ag NPs. After the encapsulation in the blends, the results of TEM microscopy for nanoparticles showed a spherical form and a dimension of 15–35 nm. Since the size of nanoparticles and their antibacterial effect are dependent properties [22], this method of encapsulation is a promising result in tissue regeneration applications [21]. Another strategy in composites is based on the development of polymeric hydrogels loaded with silver nanoparticles [23,24,25]. The variety of infections produced in recent years by bacteria led to the research of alternative solutions that are easily implemented by patients. Hydrogels based on natural or synthetic polymers are a solution in this regard since they act as a matrix for Ag NPs-targeted delivery and as support for skin regeneration. A wound dressing based on poly(vinyl pyrrolidone)/alginate/chitosan developed by Khampieng et al. [26] through the gamma radiation approach proved to have superior properties compared to commercial hydrogels. Its antibacterial effect was tested on the most common bacteria in the case of ulcers, and the results confirmed that a higher concentration of Ag NPs will increase their bactericidal activity [26].

- *Ag-inorganic composite materials*

Inorganic nanoparticles are known for their specific properties. This type of nanoparticles is divided into metal nanoparticles and metal-oxide nanoparticles. Metal nanoparticles are obtained starting from a metal source. There is a large variety of metals that can be processed in order to achieve a nanometer scale: silver, gold, aluminum, copper, iron, zinc, cobalt [27,28]. Depending on their source, metal nanoparticles have properties such as antimicrobial activity, optical activity, specific brightness [16], conductivity, surface electric charge, and crystallinity [27]. They can be obtained in different forms depending on the synthesis method and on the reagents used in it. For example, in the case of Ag NPs, the form depends on the precursors used in the synthesis method chosen. Bars, rods, spherical nanoparticles or cubic particles are obtained by choosing certain synthesis conditions and certain reducing agents [29]. In most cases, metal-oxide nanoparticles are fabricated in order to achieve superior properties as compared to metal nanoparticles [30,31]. Adding oxygen in the case zinc (ZnO) will increase its stability, while in the case of Fe_3_O_4_, its reactivity will increase [27]. In the case of magnetite, the state of Fe^2+^ from its structure allows a higher magnetite character by acting as an electron donor. In this state, such particles have a strong magnetic behavior and they are promising for magnetic field-related applications, favoring the iron metal [30]. They are used in medical imagistic therapies and in drug-releasing strategies in cancer treatment. Composites based on Ag and metal-oxide materials are intensively studied, since they offer strong resistance in addition to an antibacterial effect. If the organic composites are preferred for soft tissue restoration, in the case of inorganic materials, the applications usually aim to repair or to improve the hard tissues. Bensalem et al. [32] performed a low-temperature synthesis of a composite based on Ag-ZnO-HAp using very small concentrations of solutions for bone regeneration. The nanosized hydroxyapatite embedded with Ag proved to possess an antibacterial effect against the bacteria studied even at a very small ratio of Ag. As in the case of the other studies maintained, once the concentration of Ag is increasing, the inhibition zone is also higher [32]. The improved properties of Ag beside ZnO were also proved in the study performed by Dumbrava et al. [33], where the antibacterial effect was enhanced after the nanocomposites were obtained. Nanocomposites based on Ag-TiO_2_ films are another approach used for Ag nanoparticles. Yu et al. [34] showed that Ag increased the photocatalytic activity of TiO_2_ so that the bactericidal effect of the composite could be confirmed under UVA illumination. The introduction of Ag NPs in the matrix of TiO_2_ increased the bacterial killing rate in the case of *E. Coli* at almost 100% compared to single TiO_2_, where the percentages were around 60% [34].

## 3. Silver Nanoparticles Particularities Related to Shape, Size, and Surface

Nanoparticles are found in different forms depending on the manufacturing process. Several factors such as temperature, pressure, concentration, or time in the manufacturing process are essential in achieving the expected properties. Size, shape, and surface are properties that define the final application and destination of nanoparticles.

The shape, the main property described for nanoparticles, influences the surface-to-volume ratio and the distribution of these particles in the body. In the case of Ag NPs, the shape is a property also able to modify the bacterial response. The large surface of such nanosized particles is beneficial for drug delivery applications, cancer therapy, tissue regeneration, or photocatalytic applications [35]. Able to be encapsulated with different types of drugs or to be covered by them, nanoparticle-based materials can penetrate the body barriers to reach a selected targeted area. In the case of human or animal cells, the spherical form is preferred in many biomedical applications. Since they are integrated in biomaterials, the prevention of side effects should be taken into consideration when the shape is chosen. A large area-to-volume ratio and a right shape can lead to the proper distribution and diffusion of nanoparticles in organs, and can prevent the cell apoptosis around the tissues [35]. However, in the case of bacteria, there are literature studies which sustain the use of the triangular shape of nanoparticles, since they exhibit a high killing rate. Being able to penetrate the bacteria membrane with three edges, Pal et al. [36] suggested, through TEM images, that truncated triangular nanoplates were the most aggressive, leading to the highest capacity to kill bacteria [36]. This theory was also analyzed by Vo et al. in 2019 [37]; by comparing the spherical and the triangular shape of Ag NPs, a higher bactericidal effect was associated with the triangular shape. Previous studies attributed this result to the crystallographic structure of Ag; the (111) facet found in a higher quantity in triangular shape can interact easier with bacteria membrane and can produce necrosis faster [36,37,38]. Even if in the case of bacteria species, sharp nanoparticles exhibit stronger bactericidal effects, for a faster regeneration of the lesions and for a proper distribution in the body fluids, spherical nanoparticles are also preferred in most applications, being the most studied nanoparticles.

Size is another important property for nanoparticles in correlation with the shape and the surface [39]. The small size allows the particles to flow in the circulation system and to arrive at the injured place if they are internalized in the body. In the case of external devices, for example for burns, the small dimension of particles assures a higher penetration of them through the skin. In order to reach the tissue targeted, nanoparticles should be under or around 100 nm. Another aspect related to the size is their effect, as was described in the examples proposed. It is proved that for Ag NPs, their antimicrobial effect increases with the decrease of their dimension [40]. This observation has been confirmed in the case of magnetite (Fe_3_O_4_) [41,42], as well, where the smaller size of nanoparticles assured higher biocompatibility on breast cancer cells [43]. A similar size-dependent effect was also described by Osonga et al. [44], which compared the antifungal activity of Ag NPs synthesized with luteolin tetraphosphate (LTP) versus Au NPs synthesized with the same reducing agent. The study was performed on different types of fungi, as well as on Gram-negative or Gram-positive bacteria. After a period of incubation, a concentration of 10 μM of LTP-Ag NPs proved to suppress almost totally the growth of fungi, while at 4 μM, continuous growth could be identified. Apart from the concentration importance, the size also proved to be essential. The smallest particles identified using TEM images (around 9 nm) showed total inhibition in the case of fungi and bacteria, while at higher dimensions, the antibacterial activity decreased [44]. As well as the antibacterial effect, the size of Ag NPs was studied in the fabrication of the polysulfone ultrafiltration membrane. In a recent study of Prihandana et al. [45], Ag NPs in three dimensions were integrated in the membrane phase inversion fabrication method in order to observe the effects on the pore dimensions and the hydrophilicity of the final material. The contact angle analysis performed showed that, by comparing the control with the three membranes based on Ag NPs, the hydrophilicity is improved when the smallest nanoparticles (20 nm) are integrated. The size-dependent aspect was also proved in the evaluation of the antimicrobial activity of the membranes, where Ag NPs at 20 nm and 40 nm showed better bactericidal effects compared with the ones found in 90–210 nm [45].

The last important aspect of nanoparticles properties is related to their surface. In most cases, the surface of nanoparticles is important both in the coating process and in targeted applications. This property is related to their reactivity [46], since the Ag ions can easily create electrostatic interactions with other molecules or compounds. In the case of bacteria, the supposed mechanism of suppression is also correlated with the affinity of silver for the sulfate group, which is found in proteins or enzymes at the bacterial membrane level. The level of liquid that can be uptaken by nanoparticles also depends on the chemical surface found on the exterior of the particles. A hydrophilic structure, achieved by coating them with a high hydrophilic polymer, is usually desired in drug encapsulation. In order for the drug to be released, the nanoparticle should react in a liquid medium as it is found in tissues. Another advantage of the hydrophilic structure is the ability of nanoparticles to move away from macrophages without being destroyed [35].

Huang et al. [47] developed a facile method to combine the Ag NPs with curcumin using a core-shell structure. Through the self-assembly process, the Ag ions can be absorbed in the surface of polymeric micelles, while curcumin can be entrapped in the core. The results of the study proved that this approach has a stronger bactericidal effect on Gram-positive and Gram-negative bacteria compared to silver or curcumin used individually. In this case, the positive charge of Ag ions was involved in the electrostatic interactions performed within the templating process. Using a negative shell based on poly(aspartic acid) for the polymeric micelles, the Ag ions are easily stocked in the micelles, so this type of functionalization can assure the desired effect. Another approach is based on the deposition of Ag NPs on Ti-based materials. Titanium materials are inert and hydrophilic, however, in dentistry for example, the need to prevent possible infections and to increase its antibacterial activity led to the integration of inorganic nanoparticles such as silver. Once the Ag NPs are found at the surface of the implant, due to their high reactivity, the ions will be released and will react with sulfur or oxygen from the bacteria structure. Hajdu et al. [48] suggest that the electronic charges of Ag NPs can modify the hydrophilicity of the implant in order for the adhesion of proteins involved in the healing process to be added faster. The study also showed that the smallest Ag NPs used (around 60 nm) are presenting the most promising result in biofilm development prevention [48]. Hajtuch et al. [49] performed an interesting comparison between three types of functionalization of Ag NPs in order to observe their activity and influence on platelets function, for applications in the circulatory system. Glutathione, lipoic acid, and polyethylene glycol were conjugated with Ag NPs through chemical reactions. FT-IR spectroscopy and TEM results proved that functionalization was properly achieved in case of each reagent while the nanometric sized was maintained. Using the test based on LDH enzyme quantification, the three functionalized materials didn’t seem to produce cytotoxicity on platelets since no enzyme release was recorded. The results showed that functionalization can increase the biocompatibility of Ag NPs and coating them can limit proteins aggregations [49].

These three important aspects of Ag NPs are essential to be considered once the biomedical application is established. Depending on their destinations, Ag NPs can improve the properties of organic or inorganic materials, while maintaining their main activity of killing fungi or bacteria.

## 4. Silver Nanoparticles—Overview and Synthesis Methods

In the last few years, many synthesis nanoparticles methods were proposed. Starting from aerosols [50], an administration through a gas delivery and continuing with classical methods such as chemical or physical synthesis, nanoparticles were obtained for biomedical applications under different conditions. Solvent evaporation, emulsification, biosynthesis, precipitation, and dialysis are only a few methods through which nanoparticles can be fabricated [51,52].

Silver is a soft, shiny metal [53] used for many years in medical applications due to its benefits. Evidence described in history showed the presence of silver in ancient Egypt and Rome, being considered an excellent storage material [10]. Since the time of Hippocrates, silver was used in wound healing, ulcers, and infections. Silver began to be used either by external administration or by ingestion in order to heal intern infections [10]. In Indian culture, silver was integrated into creams or local drugs for its reaction to bacteria. For a long time, silver exhibited a strong antibacterial character being used in wound healing and prevention from diseases. In the late 1900s, treatments based on colloidal nanosilver against infections proposed by the United States led to a deeper investigation of silver-based materials used nowadays, as well [10]. The scientific and technological evolution led to the discovery of silver nanomaterials, which exhibit improved properties. Silver nanoparticles possess a high large-surface area volume which increases their activity inside the human body. The higher surface allows a better exposure to bacteria, therefore the antibacterial effect is higher [54]. As well as this, once the nanometer scale is achieved, silver particles exhibit better optical and physical properties as compared to the bulk size. Their thermal conductivity, which is the highest among metals, led to the integration of silver nanoparticles in electronic devices [55]. Their unique properties made them an interesting subject in biomedical applications, as well, where they are intensively studied for antimicrobial applications.

Silver nanoparticles are described as nontoxic antibacterial agents with strong suppression effects on a large spectrum of bacteria and fungi in infection treatments [56,57]. However, the mechanism through which they express such a strong antimicrobial effect was proved to be dependent on the physicochemical properties. Assuring small dimensions and concentrations, the immune response could be stimulated through these nanoparticles, while they would not affect the surrounding tissues. Until now, the mechanism of Ag NPs was generally described by hypotheses. The most important mechanism taken into consideration is the disruption of the cell wall. Ag NPs have the ability to penetrate the bacterial membrane through chemical interactions with phosphorous and sulfur, bases found in the proteins that surround the bacteria [22]. The lipidic structure of microorganisms is affected, so the properly transport of enzymes and proteins inside the bacteria is damaged [58,59]. The bacteria are altered in this way, while morphological modifications begin to appear due to the strong antibacterial effect. The mitochondria and the ribosomes are first damaged after the membrane penetration, while the proteins from inside the cell and around the DNA begin to decompose due to silver reactivity. Ag ions react with DNA bases while replication is inhibited, transcription and translation processes are modified, protein synthesis is stopped, and interruptions in cell divisions appear [59,60,61]. Over the years, the mechanism was proved using sensible microscopy techniques. Chwalibog et al. [62] studied the interactions of Ag NPs with *Candida albicans* and *Staphylococcus aureus* using TEM microscopy at 200 KeV. The results showed Ag NPs attached to the internal substances of bacteria after the membrane is destroyed. The disintegration of the cell wall and the release of substances were identified through TEM images, so Ag NPs proved to be toxic for both bacteria [62]. España-Sánchez et al. [63] studied, through the HRTEM technique, the contact realized between spherical Ag NPs and *Pseudomonas aeruginosa* bacteria. The images proved that Ag NPs agglomerate at the bacterial wall, eventually causing disruption and internalization. The diffusion of Ag NPs is increased within this penetration so that bacterial death is completed [63]. This mechanism seems to be more expressed in the case of Gram-negative bacteria. The differences in cell membranes between Gram-negative and Gram-positive bacteria are based on the wall composition and thickness, so the thin layer of peptidoglycan found in Gram-negative bacteria is more suitable for the antibacterial effect of silver [56,59]. Pazos-Ortiz et al. [64] developed a nanocomposite based on Ag NPs embedded in poly–epsilon–caprolactone nanofibers using the electrospinning process. DLS measurements showed a nanometric scale of 10–15 nm, while the SEM images confirmed the internalization of Ag NPs in the polymeric fibers. The antibacterial analysis was performed comparing the Gram-negative to Gram-positive bacteria. It was proved that, in case of *E. Coli* (Gram-negative bacteria), the inhibition zone was higher comparing to *S. aureus* (Gram-positive bacteria) at the same concentration of Ag NPs. At 100 mM of Ag NPs, the inhibition zone of *E. Coli* was recorded at 39.36 mm, while in the case of *S. aureus*, the inhibition zone was established at 34.63 mm. The study also showed the gradual increase of the antibacterial effect while the Ag NPs concentration is increasing [64], so the dependence between Ag properties and the final effect is one again confirmed.

Silver nanoparticles are extremally versatile regarding their applications. Over time, they have been used in medical applications, typically for antibacterial actions, in house cleaning, in coatings, and even in solar energy [65]. In medical applications, they are integrated in hydrogels, can be coated with natural compounds, can be deposited in metal surfaces for infection preventions, and can be mixed with drugs for specific targeted applications. Silver nanoparticles also have antiviral properties, leishmaniasis activity, anti-diabetic and antioxidant effects, since they are involved in ROS (reactive oxygen species) activation [66]. These free radicals are hardly reactive species, which can increase the effect of Ag NPs. Integration in biomaterials is the most studied approach, since single Ag^+^ found in a high amount can quickly oxidate and can induce damages or cytotoxicity. However, in all medical applications, their activity remains the same, assuring an antibacterial activity for possible infections. A novel nanogel based on chitosan was developed by Fan et al. [67] to ensure the slow-release of Ag and to prevent possible damages to surrounding tissues. Using the electrostatic interactions between sulfonated chitosan and chitosan/Ag NPs, nanogels with Ag^+^ absorbed were obtained. The study presents an interesting comparison between short-term activity and long-term activity and the biofilm ablation of these nanocomposites on *E. Coli* and *S. aureus* species. The term-long activity was tested after the samples were maintained for long periods in 7.4 pH PBS, similar to the tissues pH. The results showed a very good antibacterial effect (around 99%), even after 6 days of incubation, so the results are promising for a long-time inhibitory effect. It was proved using the CFU counting method for both bacteria species and for both times analyses, that the survival of microorganisms decreases while the incubation time and the concentration of composites are increasing for short-term analysis. Moreover, the study confirms the differences observed in other studies described between Gram-negative and Gram-positive species, since the effect on *E. Coli* was obtained faster and in lower concentrations (150 μg/mL in 90 min) comparing to *S. aureus*, where even at a double concentration, their action was delayed (300 μg/mL in 210 min) [67].

Another application of Ag NPs in wound infections can be represented by nanosheets. Easy to use, as in the case of hydrogels, they are a promising solution for burns or lesions accompanied by infections in order also to increase tissue regeneration. Yuwen et al. [68] fabricated a functional MoS_2_-polydopamine-Ag nanosheet for *S. aureus* infections. The sheet based on MoS_2_ was first obtained through an innovative lithium intercalation method, and after the fabrication it was coated with polydopamine for a better protein adhesion of the final material. The chemical structure rich in phenolic and nitrogen-reactive groups of the polydopamine absorbs Ag^+^ to form Ag NPs. The diffractions obtained through XRD showed the diffraction peak associated with the (111) plane in the case of Ag, so the absorption proved to be a successful one. Moreover, X-ray photoelectron spectroscopy confirmed the presence of Ag, since two specific peaks for Ag 3d_5/2_ and Ag 3_d/2_ were recorded [68]. The analysis performed on *S. aureus* biofilms proved, once again, the reduction of bacterial film biomass when the bacteria are incubated with Ag-nanosheets [68].

Silver nanoparticles can be synthesized through different methods depending on the application that they are required for. Over the years, different chemical or biological reagents have been studied in various reactions. The technology, the reagents, or the parameters such as temperature and flow rate led to the fabrication of silver nanoparticles in various conditions. Physical, chemical, or biological methods are used in almost all cases [69,70,71,72]. The main synthesis methods are presented in Table 1.

### 4.1. Chemical Methods

Chemical synthesis is used in most research studies. The advantages of such techniques are well-known in literature studies. Affordable equipment, high yield in the manufacturing process, ease of handling, and a variety of reagents which can be used are the main factors that determine this choice. A possible issue regarding this method is related to the shape control of the nanoparticles obtained. To maintain the spherical shape of silver nanoparticles, as it is required in most biomedical applications, certain conditions must be accomplished. The most important factors are related to the reagents chosen and to the temperature used in the synthesis process.

#### 4.1.1. Chemical Reduction

**Figure 3:**
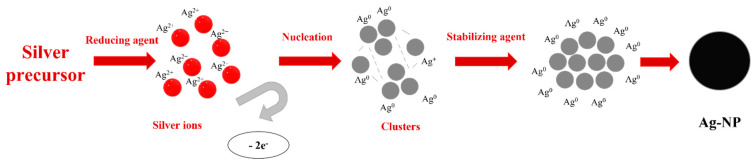
Mechanism of chemical reduction of a silver precursor in Ag-NP formation.

Chemical reduction is based on a metal salt source which is reduced with reducing and stabilized agents. Among different silver sources, silver nitrate (AgNO_3_) and silver perchlorate (AgClO_4_) [69] are the most used. Reducing agents have an important role in this method due to their correlation with nanoparticle shapes. Glucose (C_6_H_12_O_6_), ethylene glycol (C_2_H_6_O_2_), and sodium borohydride (NaBH_4_) [69] are reducing agents which act in the reduction of Ag^2+^ to Ag^0^ during the process. These reagents involve the transformation of precursors in metallic silver (Ag^0^), which will induce the formation of clusters by agglomeration. The clusters are further grouped to form colloidal silver nanoparticles [81]. To be maintained and perform the reaction in the best conditions, stabilizing agents must also be present. They have the role of stabilizing the nanoparticle’s suspension while nanoparticles are formed and avoiding a possible agglomeration of particles. The mechanism of such reactions is depicted in Figure 3.

The research studies on silver nanoparticle synthesis performed over the years led to the establishment of a connection between agents and the form of nanoparticles, as illustrated in Table 2. Pyrrolidone (PVP), polyethylene glycol (PEG), or polyvinyl alcohol (PVA) can be used in this regard [14]. In the reaction described above, the silver source can be chosen as silver nitrate (AgNO_3_), since it is the most suitable precursor for Ag NPs. Using a reducing agent, the silver ions become free while they are reduced to atoms [81]. The atoms will be transformed further into clusters by a nucleation reaction. The stabilizing agent is present from the nucleation step until the end of the process. It assures that colloidal silver particles synthesized will not agglomerate and a nano-size will be maintained.

Sodium borohydride is the agent used in the chemical reduction method for silver nanoparticle synthesis. Its main advantage regarding its use both as a reducing agent and as a stabilizing agent can be controlled by the special synthesis conditions that are required. In a recent study [76], a group of researchers obtained silver nanoparticles using sodium borohydride as the main chemical agent. Their experimental setup started from a silver nitrate solution and by using a 0.02 M solution of sodium borohydride solution on an ice bath, they induced the chemical reaction for Ag NPs synthesis. A cooling system is needed in the case of sodium borohydride. Avoiding both a possible second reaction and a possible reaction with the nitrate ions resulting from a high temperature are conditions required for using sodium borohydride. The researchers found that using the ice bath could overcome these disadvantages, therefore such experiments require a cooling system. The results of the study revealed that nanoparticles were obtained avoiding the aggregates production. The SEM images and X-ray graphs determined approximately a 30 nm dimension for Ag NPs, thus proving that the size can be influenced by the reaction conditions and by the quantity of sodium borohydride [76].

A comparative study published in 2019 by a group of researchers from Costa Rica [84] strengthened the idea of proportionality between the conditions of reactions and the particle size. Another route regarding silver nanoparticle synthesis and sodium borohydride is based on the use of PVP in reaction. Poly(vinyl) pyrrolidone is a well-known stabilizing agent in such synthesis. The method involving PVP led to nanoparticles with dimensions between 15–30 nm in various shapes. Parameters such as the concentration of PVP or its molecular weight change the size and shape of nanoparticles. Song et al. [88] dedicated a research study on the influence of the molecular weight of PVP in silver nanoparticle synthesis. The results revealed that PVP can play a key role in controlling the shape of the particles. Using a PVP with a small molecular weight (MW = 8000), the microscopy analysis revealed the formation of nanorods joint together at one of the ends. A molecular weight of MW = 29,000 led to nanoparticles formed in a spherical shape. This molecular weight seems to perform the most uniform silver nanoparticles at a dimension around 60 nm. The next molecular weight studied, MW = 40,000, caused the synthesis of non-uniform shapes of nanoparticles. Some wires could be identified, but in a high percentage, the SEM images proved that the shape is irregular. At the highest MW analyzed MW = 1,300,000, the nanoparticles were obtained in a wire shape. In the SEM images obtained at a 500 nm scale, the wires are clearly identified as preponderant shape [88]. This interesting study proves that for silver nanoparticles synthesis the parameters of reactions are key elements in the shape control. This aspect is actually important in biomedical applications since the cells must not be affected by the sharp edges of the biomaterials. A similar form as the one of the cells can assure the success of the patient’s recovery over time.

The conditions of chemical reactions can influence not only their shape but the size, as well. Another study proved that nanoscale particles are obtained using certain reducing agents and stabilizing agents. Zielinska et al. [83] proposed the synthesis of silver nanoparticles using ascorbic acid, hydrazine and sodium borohydride as reducing agents and PVA or PVP as stabilizing agents. The results revealed that using PVP as stabilizing agent together with ascorbic acid or hydrazine as a reducing agent led to nanoparticle dimensions between 30–80 nm. On the other hand, PVA used as a stabilizing agent led to some smaller particles, the range of dimensions being between 44–48 nm. The biggest particles were identified when sodium borohydride was used in the chemical reactions [83]. These experiments revealed that beside the extreme conditions that are required, sodium borohydride presents the inconvenience of producing bigger particles.

Silver nanoparticles exhibit optical properties, among others. The optical properties are used in their characterization to determine their shape and distribution. Information regarding their physical behavior can be determined by analyzing the spectral properties of these nanoparticles in solution. Silver particles absorb and scatter light due to their state. Subjected to a light with known wavelengths, the electrons from their surface start to oscillate. In this way, the particle can absorb and scatter a certain amount of light identified by UV-VIS spectroscopy [89]. These properties were studied in a research paper in which silver wires were identified [86]. The use of AgNO_3_ and PVP in reaction revealed that silver nanowires with diameters in the range 30–40 nm were obtained [86].

#### 4.1.2. Micro-Emulsion Technique

**Figure 4:**
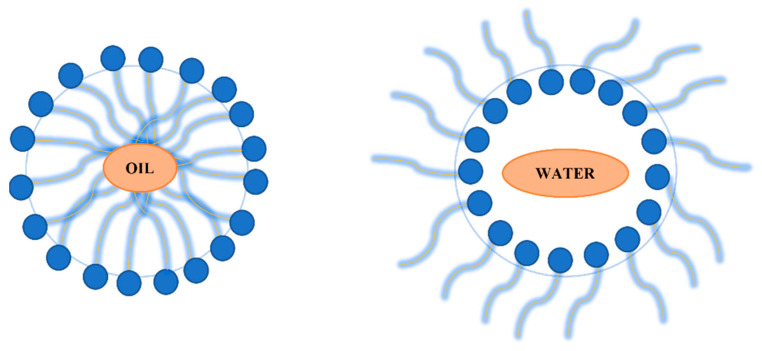
Oil in water micro-emulsification versus water in oil micro-emulsion.

Microemulsions showed interest in silver nanoparticles synthesis over time. The possibility of producing uniform and controllable sizes of silver nanoparticles led to the investigations of this method. Microemulsions are isotropic solutions with good homogeneity and thermodynamically stable and made up of three components: a polar phase (water), a nonpolar phase (oil) and a surfactant [90,91]. Their low interfacial tensions, large surface area, high stability and capacity to work with immiscible agents are advantages taken into consideration in the nanoparticles synthesis [92]. The surfactant is found between the polar phase and the non-polar phase forming a “layer” observed at the microscopic scale. With the surfactant found at the interface of the two layers, microemulsion can be made in two ways: drop by drop of oil in a continuous water phase, known as oil–water micro-emulsification or droplets of water in a continuous oil phase, known as water–oil micro-emulsification. Differences can be observed in Figure 4 where both methods are represented [90].

The micro-emulsion technique consists of different steps. Initially, a separation of the metal precursor and of the reactive agent into two phases that are not mixing is carried on. The interaction between these two reagents, metal source and reducing agent, is mediated by an ammonium salt at the interface. The interaction rate between the metallic precursor and the reducing agents is influenced by the interface that appeared between the two liquids and by the intensity of the transport between the two phases. The formed metal clusters are stabilized by the stabilizing reagent introduced in the reaction and transferred in medium by the organic solvents. During the final step, both the surfactant used in reaction and the organic solvents will be removed from solution by ultrasonication and the particle will be separated [90,93]. The main disadvantage of such a method is related to the organic solutions used. Even if the micelles used can offer a controllable size effect, the use of organic solvents is considered to have a negative effect if the nanoparticles are intended for biomedical applications.

To use eco-friendly materials in microemulsion method, Das et al. [77] used in Ag NPs synthesis a biosurfactant, rhamnolipid, extracted and purified from *P. aeruginosa* bacteria. Starting from bacteria cultures, the biosurfactant was extracted using ethyl acetate which was further purified using a silica column. The study integrated eight colonies of microorganism extracted and the most suitable one was chosen as biosurfactant in Ag NPs synthesis. TEM images showed a spherical form for nanoparticles with good dimensions found between 2–30 nm. The antibacterial analysis realized through the test of inhibition revealed that zones exposed that Ag-NPs presented a necrosis effect for all bacteria species studied. Their action is higher in the case of *S. aureus*, results which correlate with a high number of literature studies [77].

#### 4.1.3. Electrochemical Synthesis

Another alternative strategy in silver nanoparticle manufacturing is electrochemical synthesis. This approach is based on a two-electrode system with a cathode and an anode. The main advantages of such a technique are related to the controllable size of the particles and to the possible modifications that can be applied to the current density used. Even it seems a promising method, in the case of biomedical applications a possible disadvantage lies in the fact that organic solvents are required [94]. In 2015, a group of researchers proposed a basic electrochemical synthesis in which stable silver nanoparticles were obtained with simple compounds in reaction. The reaction was performed at the interface of a solution based on an AgNO_3_ solution. The solution and the carbon electrode were placed in a cell and the nanoparticles were obtained for a certain current density. The results revealed that once the silver ions are found in a high concentration, aggregation can appear. X-ray results revealed a 25–45 nm dimension for the particles, while the SEM images showed a dendritic form of the particles [94].

A group of researchers from Romania [78] proposed an electrochemical method called „sacrificial anode” based on stabilizing solutions for nanoparticles. The method proposed is based on an Ag electrode immersed in dispersing medium and on a mixture of stabilizing solutions. The formation of colloidal silver was reported with a 5–10 mA current for a time range between 3–7 h and with PVP 550,000 and Na-lauryl sulfate as stabilizers for Ag NPs. The results of the study proved that the use of stabilizers can induce stable particles without agglomeration. DLS measurements and microscopy images revealed a spherical form of nanoparticles with dimensions between 10–55 nm. Such results are encouraging for biomedical applications since no organic solvents were used. Even if Na-lauryl sulfate is an ionic solution, the purity of silver nanoparticles is assured during the process [78].

Electrochemical synthesis is a promising method for silver nanoparticles synthesis. As it was proved already, each method can assure different sizes and shapes of particles, therefore controlling the parameters in the reaction is essential. A certain kinetic of reaction can hinder the possible agglomeration of particles, therefore their formation in aqueous solutions must be controlled by the cathode reactions [95].

#### 4.1.4. Hydrothermal Method

The hydrothermal method is one of the most studied methods in case of nanomaterials due to its controllable conditions. Based on a chemical solution, the reaction in which nanoparticles are obtained is made with low or high-pressure and temperature depending on the morphology expected. The yield of the reaction through this method is high, so with this hydrothermal method the loss material is the smallest [96]. The principle of the method is based on the differences of temperature appeared at a high pressure which favorites the inorganic solution or the insoluble materials. Once the reagents and the water are incorporated in the system, the formation of ions begins in a steel pressure enclosure. With a kettle from the equipment, the ions are separated and conducted to a low-temperature region where the crystals form in various morphologies depending on the condition established before the experiment [91,97]. The silver nanoparticles can be obtained within this method based on their nucleation reaction used in all the chemical synthesis methods.

Wang et al. [79] used the hydrothermal treatment to load the Ag NPs on a TiO_2_ surface. The doped surface with Ag NPs obtained proved to have the same porous morphology, so no alteration was produced during the process. The SEM images showed a growth in grain size dimension in the titanium surface once the silver solution concentration used is increasing. The presence of silver was also proved through X-ray photoelectron spectroscopy (XPS) by identifying the metallic silver in three bonding states (Ag 3d; Ag 3d_5/2_; Ag 3d_3/2_). Since the applications of this study was related to orthopedic field, the antimicrobial tests were performed on the *S. epidermidis* and *S. aureus*, the most frequently bacteria appeared as biofilms in surface implants for bone infections. The results showed a stronger bactericidal effect while the concentration of Ag is increasing. Starting from 7.3 CFU/mL for *S. epidermidis* in case on TiO_2_ and achieving a 2.7 value for samples with the highest wt.% of silver is a strongly suggestion that such materials can stop the bacterial colonization on the surface of an implant. The results are similar for *S. aureus*, so the prevention of bacteria adhesion was also smaller [79]. Another interesting application of Ag NPs through this synthesis method is their activity in gene delivering since the proper hyperbranched polymer is chosen. Liu et al. [98] obtained Ag NPs functionalized with polyethyleneimine using this chemical route at different temperatures. Keeping the same concentration for solutions, the synthesis was performed at 100 °C, 140 °C, 160 °C and 180 °C since many literature studies sustain that nanoparticles shape is a temperature-dependent property. TEM images showed that in the lowest temperature, the nanoparticles are found in a spherical form, while at the highest one, they begin to achieve a triangular and rod shape, so the influence of parameters was confirmed once again [98]. Oscoy et al. [99] proposed, for the first time, the synthesis of Ag NPs through hydrothermal technique using red cabbage extract. The study results showed that the combination of green chemistry and hydrothermal method can achieve the antibacterial effect. An attractive affirmation of this results are the modifications in charge density at Ag. The reactive groups found in red cabbage bond to the surface of Ag NPs and lead to a negatively charge of silver according to zeta potential analysis [99]. This characteristic can reduce the aggregation process, so Ag NPs will remain longer in a stable phase which is a very important aspect in biological application. Since their mechanisms of elimination is not entire known, the reduction of possible effects is imperative necessary.

### 4.2. Physical Methods

Physical methods in silver nanoparticles synthesis gained researchers attention lately especially due to the controllable parameters used in reactions. In comparison with chemical synthesis or biological methods, the physical approaches do not need solvents or reagents that could contaminate the samples. The uniformity of particles is theoretically higher if they are produced by physical methods. Even so, the need of expensive and large equipment, of long time to prepare the devices and to achieve results or the need for controllable parameters (for example, temperature) are inconvenient hard to overcome [93].

#### 4.2.1. Laser Ablation

Laser ablation is the most common method for Ag NPs synthesis among the physical approaches. There are some important parameters that must be taken into consideration: the laser wavelength, the force the metallic target is bombarded with, the laser pulses, the ablation time or the existence of surfactants [93,100].

The laser ablation method uses a laser to irradiate a solid material in order to be reduced to particles of nanometric dimensions. The material is exposed to laser irradiation for further fragmentations in forms of particles. Different types of lasers can be used, one example is based on Nd:YAG (neodymium-doped yttrium aluminum garnet) [101]. The fragmented particles remain in the solution found around the target and the colloidal solution is obtained after many laser pulses. The parameters established before the experimental setup can influence the final concentration and state of nanoparticles solution [101].

In a recent study, Rafique et al. proposed a comparison between two types of lasers in silver nanoparticles synthesis by ablation [73]. The Nd:YAG common laser with a wavelength of 1064 nm and pulse length of 10 ns was compared in a research study with a CW diode pumped solid state laser with a wavelength of 530 nm and a power of 10 W. The Ag target was cleaned and placed in a quartz cuvette while it was bombarded with each type of laser source to obtain colloidal silver nanoparticles. The results of the study proved differences between the two methods performed. AFM images showed that a spherical form was obtained in both cases, but the dimension is higher with the CW laser. An increase in sizes (4–14 nm versus 10–30 nm) was correlated with the oxidation and surface charge around the particles. Beside the microscopy aspect, the thermal conductivity was also proved to be higher in case of laser pulse. The thermal conductivity of Ag-nanofluids was tested by TCLGU technique and showed an improvement of thermal conductivity with 11% in case of pulsed laser compared with CW laser. The correlation between results proved that the pulsed laser, the traditional method, remained superior to the other laser sources [73].

The technique has some impressive advantages in particle synthesis. The first is the possibility of producing a high number of particles in a single process. The second one is related to the capability of the laser source to generate different concentrations of colloidal solutions depending on the parameters set [101]. Even if it is a promising method, the main disadvantage remains the expensive and large equipment needed. The time of analysis is higher compared to chemical methods and the process is more complicated.

#### 4.2.2. Ball Milling

Ball milling is a mechanical method used to produce nanoparticles. It was mentioned first in 1970 and this method is used nowadays especially for metallic and ceramic nanoparticles. Its main advantages are related to the low cost, the ability to process hard materials, the small dimension of particles obtained and their purity since no solvent or chemical reagents are needed [101,102].

This method requires a high force applied to activate the electrons from the crystals of the material and to activate the electrons from the inner structure. Compared with a higher temperature of 1000 °C, this technique is able to grind the material in a better manner. The difference of the velocity between the balls with rotational movements and the grinding jars lead to the release of high energies in the process. The numerous collisions will induce the high temperature required to initiate the chemical synthesis. This high temperature is involved in atom diffusion of atoms during the process, therefore the formation of nanoparticles or doped nanoparticles is achieved [102].

A study perfumed by a group of researchers from Slovakia showed that ball milling is also suitable for the green synthesis of silver antibiotic, a strategy intensively studied in the last years. The silver nitrate was used in the presence of two natural compounds acting as reducing agents through the ball milling method. *Origanum vulgare L.* and eggshell membrane were used as reducing agents with the silver precursor solution. The results of the study revealed that both biocompatible compounds could be used as reducing agents within this method, but with some differences. The TEM images showed that in case of *Origanum vulgare L.* the dimensions of particles are smaller. The optical and antibacterial properties seem to be better in case of the plant compared to the eggshell membrane, as well [74].

#### 4.2.3. Ion Sputtering

Ion sputtering is a physical method based on the deposition of substrates using a high energy equipment and an ionized gas. The method is based on the introduction of argon gas which through a high electric field will create a plasma inside the cavitate and the ions will migrate from the anode toward the cathode as an accelerate focused ion beam. The focused ion beam will be further sputter on a targeted material, in most of the cases, as an adherent film. Being a top-down method, a high vacuum is mandatory so the gas ions could be accelerated and the deposition could be completed [103].

López-Lorente et al. [75] used ion beam sputtering method to deposit nanocomposite based on various fractions of Ag and TiO_x_/ZnO on silica substrates for better observation of modifications appeared in single molecule analysis such as surface enhanced Raman spectroscopy (SERS). There were prepared three samples: Ag NPs deposited on ZnO, Ag NPs deposited on TiO_x_ and a mix sample of Ag-TiO_x_ performed using two targets as a co-sputter configuration. The results of the study showed that the use of silver and ceramics increase the sensibility of substrate so deeper information in vibrational spectroscopy can be obtained using them. Acting also as photocatalytic materials, the samples based on Ag/TiO_x_ provided the highest signal enhancement in SERS. These nanocomposites have the advantage of being reusable and capable of auto-cleaning since their catalytical activity [75]. This study proved that Ag NPs have also excellent properties in chemistry and surface functionalization beside their antimicrobial effect, so their ability to the used as substrates in spectroscopy is a promising approach for further studies.

### 4.3. Green Synthesis

**Figure 5:**
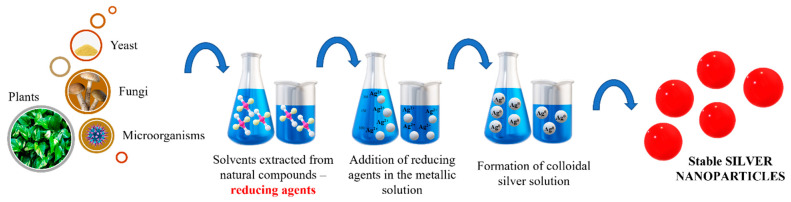
General green synthesis starting from natural compounds in Ag NPs synthesis.

Green synthesis has evolved as a strategy for silver nanoparticles synthesis. The biosynthesis of particles seems to have a smaller impact on the environment since the reducing agents are based on plants, bacteria, fungi, yeast or microorganisms. Natural compounds extracted from plant roots or various bacteria are used to reduce the metal salts used as precursors. Depending on the possibility of experimental setup and on the choice of certain equipment, other biological material can be used, as illustrated in Figure 5.

#### 4.3.1. Plants

Plants containing specific bioactive compounds are able to reduce the metallic salt used as precursor in silver nanoparticles synthesis. Some molecules found in the chemical structure of plants, flavones, ketones, amino acids, phenolics, aldehydes or vitamins, have the role of reducing the silver ions [104]. The low cost and their potential are advantages that could overcome the traditional chemical route. However, even if the green synthesis can be considered an easier pathway to produce nanoparticles, the chemical structures found in solvents could interfere with the particle’s stability. Between all types of reducing agents in green synthesis, plants are considered the safest choice. The kinetic times of reactions are higher in this type of synthesis compared to other biosynthesis methods. The phytochemicals produced by them can act faster as solvent and can modify the metal salt solution in a shorter time [105]. The process involves any part of a plant. Even if fruit, leaf or roots are chosen, the first step is based on boiling. The leaves are boiled in ultrapure distilled water and after they are squeezed, the solution is filtered. The reducing solution is mixed with the metallic precursor and after the color changes, the nanoparticles can be separated [105].

#### 4.3.2. Bacteria

Bacteria can reduce the silver ions through two mechanisms. Some existing bacteria proved a strong resistance to silver particles over time. This type of microorganisms can aggregate the silver nanoparticles in two different ways: intracellular or extracellular. The process of synthesis begins when bacteria is attracted by the ions from the solutions. The microorganisms catch the targeted metal ions and transform them to elemental atoms through enzymatic reactions. The difference between intra or extracellular is related to the location where particles are formed. In the extracellular process, the silver ions are trapped on the cell’s surfaces and reduced in the presence of specific enzymes. In the case of an intracellular process, the silver ions are transported inside the cell and the particles are synthetized there [106,107].

An important advantage regarding this approach is based on a high sustainable property. Bacteria are easy to handle organisms which can produce the particles needed in a high quantity and on a high scale. The absence of toxic chemicals is also a positive aspect of this method. However, green synthesis based on bacteria also revealed some limitations. The impossibility to control the precipitation process has a strongly negative impact. The control of nanoparticle size is not possible, therefore their distribution and uniformity are totally random [107].

#### 4.3.3. Fungi

Using fungi is also possible in the green synthesis of silver nanoparticles due to their properties. A high ability of accumulation and binding, high tolerance, and the capacity to internalize particles make them a competitor to bacteria or plants. Usually, the metallic precursor, for example AgNO_3_ is reduced by the enzyme from fungi structure. As for bacteria, the method can be performed in intracellular or extracellular space. Depending on the type of fungi used, for example *F. oxysporum* versus *Cladosporium cladosporioides*, the process of precipitation can undergo at the cell surface or inside the cell. Studies realized over time proved that size distribution is also different in those cases comparing 10 nm with 10–100 nm respectively [106].

In recent years, green synthesis was studied more and more with different compounds. The type of bioactive molecules found in its structure, and the conditions of reactions led to many shapes of nanoparticles and different sizes. Considering the final applications explored in thispaper, the spherical form is preferred, so Table 3 presents the main types of plants or microorganisms used for silver nanoparticles.

## 5. Advanced Characterization Techniques for Ag NPs

Over time, the evolution of materials science required development of advanced methods for nanoparticle characterization. Nanoparticles can be characterized with respect to their size distribution, surface, stability, morphology or interactions with other molecules. The main techniques that are currently used, depending on the properties that are investigated, are presented in Table 4.

### 5.1. X-ray Diffraction (XRD)

X-ray diffraction is a quantitative and qualitative method used for liquid and solid nanostructured materials. Its main use is the determination of crystallographic structure, but it also provides strong information about the constituent phases in the material once a nanomaterial is synthetized.

**Figure 6:**
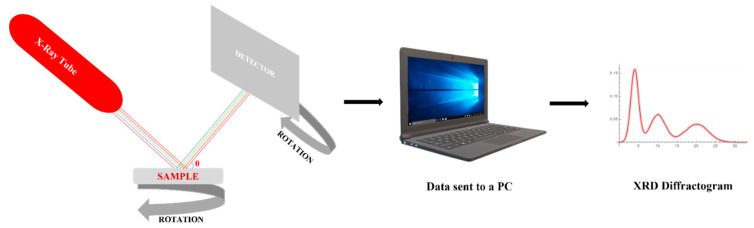
Schematically representation of XRD principle.

Figure 6 presents a schematic of the method. The X-ray tube uses an electron beam emitted by a cathode and accelerated at high voltage to the anode which is found at the sample’s surface. The sample is set on a rotative support situated in the diffraction space, so all the edges of the sample could be analyzed.

The diffraction is described by Bragg’s law, which establishes a relationship of proportionality between the wavelength of radiation λ, the reticular distance between planes, *d* found in the material’s structure and the angles *θ* between the incident X ray beam and the sample Bragg law, on which this technique is based, can be expressed through a mathematical equation:

(1) $$d=\frac{n\lambda }{2\sin\theta }$$

The Equation (1) involves different *θ* angles obtained through the analysis of results from which the distances *d* can be recorded and associated to the crystallographic planes for each element. In case of Ag NPs the XRD expectations are based on the identification of (111) plan which is associated with Ag in a face-centered-cubic structure.

The grain size of crystallites is calculated according to the Scherrer Equation (2) in which the angle *θ* plays an important role. Beside the phases and crystallinity structure, XRD can determined the average size of a crystallite by analyzing the width of a peak from the diffractogram located at an angle *θ*:

(2) $$D=\frac{k\;\lambda }{\beta /2\cos\theta }$$

In Scherrer equation, *D* is the average crystallite size, *β* is the full width at half maximum of a peak identified in the diffractogram, λ is the wavelength of radiation, *k* is a constant (0.94) and *θ* is the angle of tha particular maximum, the angle in Bragg’s law. This equation is used for dimensional analysis of the nanoparticles from the sample. The average size of a crystallite is cross correlated with the results of other microscopic techniques to establish the nanometric size of the material such as Atomic Force Microscopy, Transmission Electron Microscopy and Dynamic Light Scattering.

The crystallinity of a silver-based material can be identified through XRD technique depending on its state of aggregation. In most cases, XRD is employed on solid materials since the radiation is strong and damages can appear. Lately, suspensions were also analyzed by transforming them into solid fragments first. In a recent study, a group of researchers synthesized a silver suspension using green synthesis [120]. Using silver nitrate as source and callus extract as reducing agent, silver nanoparticles were synthesized at room temperature. XRD analysis was performed on the centrifuged solution transformed into pellets which were further dried in the oven and analyzed as solid materials. The diffractograms with θ angles between 32° and 77° suggested that nanocrystals were present in the sample. The Scherrer equation on the full width at half maximum of each peak showed that nanoparticles investigated were found in a range of 42–44 nm. The results reported for this green synthesis proved that even if organic compounds produce unassigned peaks, small particles with nanometric dimensions can be synthetized [120].

Another group of researchers [76] studied the synthesis of silver nanoparticles using sodium borohydride as a reducing agent. Based on a chemical reduction, the reaction was performed with high yield leading to the obtaining of 0.054 g silver nanoparticles from 50 mL solution of precursor. XRD analysis performed on the silver suspensions showed similar values for *θ* angles as in the most studies realized on silver particles. A maximum of 38.3° indicated on the diffractogram was used for crystallite size determination. The study results showed an average of 33 nm size, therefore the method was found suitable for silver nanoparticles synthesis [76].

**Figure 7:**
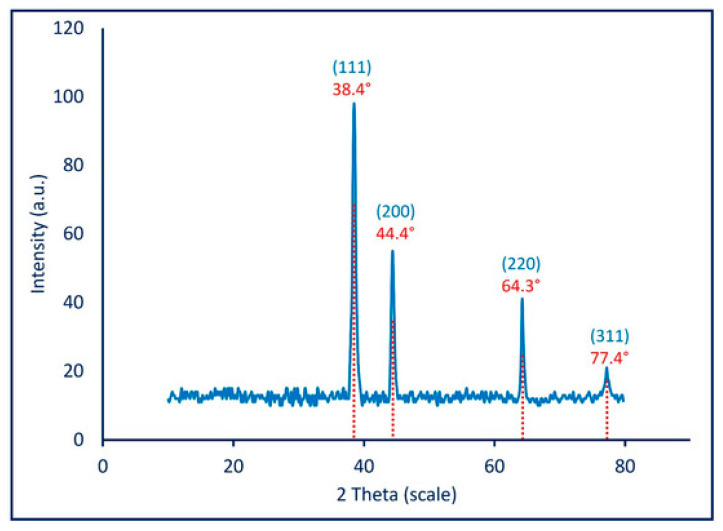
XRD diffraction for Ag NPs through mycosynthesis Reprinted from an open source article [121].

Awad et al. [121] obtained Ag NPs through a mycosynthesis method using green chemistry and *Aspergillus niger* as a reducing agent. The innovation of the study is related to the targeted microbial agents which were chosen as the eggs of microorganisms. Ag NPs obtained through this method were first tested for structural information using spectroscopy and microscopy methods. The XRD diffraction (Figure 7) shows a crystalline form for the nanoparticles obtained. The highest peak obtained is correlated to the (111) plan, also mentioned previously. The four peaks observed are associated to a face-centered-cubic structure and the accuracy of the graph suggests stable Ag NPs based on a successful synthesis [121].

### 5.2. Dynamic Light Scattering (DLS)

**Figure 8:**
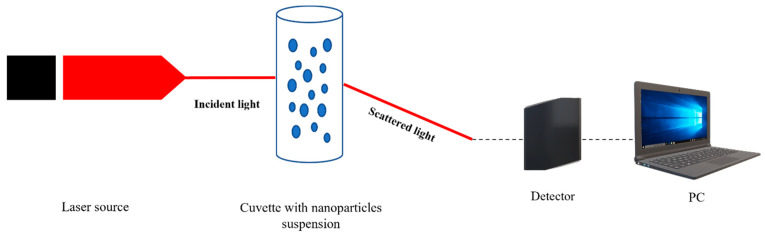
Schematically representation of DLS in nanoparticles suspensions analysis.

Nanoparticles size measurements are currently performed using an optical technique such as Dynamic Light Scattering, as well. Found in suspensions (Figure 8), nanoparticles move according to a diffusion model which can be detected and processed.

DLS is based on the Rayleigh scattering theory, and it is used in the study of nanoparticles, colloids or proteins, as is described in many papers [122,123,124]. At irradiation with a laser source, the particle from suspensions will absorb and scatter light depending on its size, as it happens in the case of Ag NPs. The detector will measure the intensity of scattered waves which will be transformed in an electric signal and further in integer number by using a data acquisition system [125,126,127,128]. Once a time series is recorded, it is processed using the autocorrelation of intensities.

The data acquisition system does not have to be special [129], and records values at equal time intervals (Δ*t*) known as time-series, as described further [124]:

I(0), I(Δ*t*), I(2Δ*t*), I(3Δ*t*),…, I(nΔ*t*) (3)

The scattering vector *q* is the difference between the reflected and the incident propagation wave vector and is described by Equation (4), where the refraction index of the solvent is *n* and the wavelength of the laser radiation is *λ* [122]:

(4) $$q=\frac{4\pi n}{\lambda }\sin\left(\frac{\theta }{2}\right)$$

The scattering vector *q* is integrated in the autocorrelation (*G*(*τ*)) function used in plotting the autocorrelation. Depending on the particles size distribution, if it can be approximated as being relatively narrow the monodisperse approach can be used. In this case, after normalization in two-steps, the autocorrelation of the light intensity can be described as an exponentially decaying curve [123,124]:

(5) $$G\left(\tau \right)=e^{-2Dq^{2}\tau }$$

The final step consists of assessing the diffusion coefficient *D* using a least square minimization procedure or by using an artificial neural network approach [130,131,132]. Further on, using the Stokes–Einstein Equation (6), the average hydrodynamic diameter *d* can be assessed:

(6) $$D=\frac{kT}{3d\pi ղ}$$

In Equation (6) *k* is the Boltzmann constant, *T* is the temperature, *D* is the diffusion coefficient and *ղ* is the dynamic viscosity of the solvent [133]. This last step will be integrated in the final size determinations of the particles found in a Brownian continuous movement in the suspension being studied.

**Figure 9:**
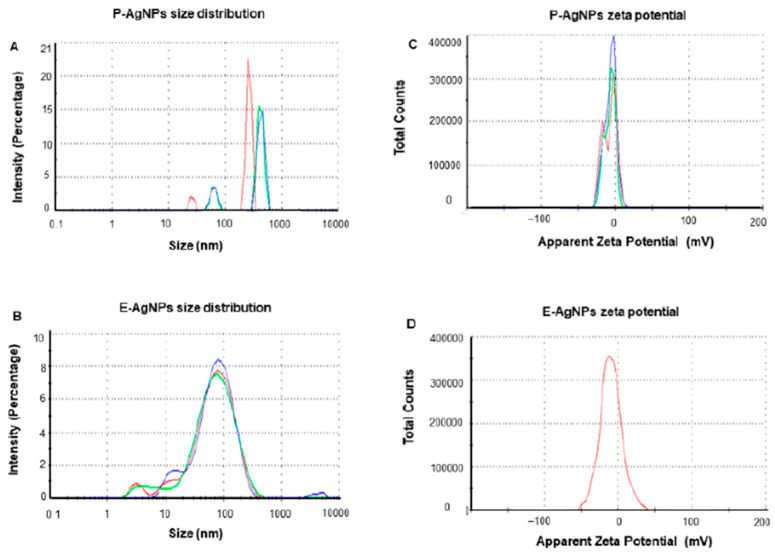
DLS analysis for P-AgNPs and E-AgNPs. (**A**,**B**): distributions for size and intensity. (**C**,**D**): zeta-potential for surface charge. Reprinted from an open-source article [134].

DLS is preferred in most studies regarding nanoparticles characterization if they are suspended in a fluid. In a recent research paper, a group of scientists form Denmark proved that silver nanoparticles synthesis can also modulate their size. The silver nanoparticles were synthesized through a green chemistry approach using two types of pathogens as reducing agents: *Pseudomonas putida KT2440* (P-AgNPs) and *Escherichia coli K12 MG1655* (E-AgNPs). They were tested on the corresponding pathogens *Pseudomonas aeruginosa* and *Escherichia coli* to observe if there are correlations between precursors used and targets in their antibacterial effect. DLS analysis was performed to determine the hydrodynamic diameter and zeta potential for the Ag-NPs investigated. In the first two graphs (Figure 9A,B) the polydispersity index (PDI) is illustrated. The results proved that Ag-NPs were found in a stable form mostly monodisperse in the solution. The E-AgNPs proved better stability due to a lower intensity. Also, regarding their dimensions, E-AgNPs were found around 47 nm while P-AgNPs were found to be around 108 nm. The size distribution for both pathogens used in the green chemistry synthesis is a promising result since they exhibit nanometric dimensions. The zeta potential (Figure 9C,D) completed the measurements and revealed that these nanoparticles are stable in the solution due to the high negative values they had. The study proved that the synthesis method can strongly influence nanoparticles size distribution and efficiency regarding their antibacterial properties [134].

The hydrodynamic diameter is related to the way particles are diffusing. Nanorods, nanocones and particles of any shape or form are diffusing in a solvent and DLS has as output the diameter of the spherical particles that are diffusing at the same rate as the particles being investigated. Atomic Force Microscopy is a complementary technique that can be used to investigate the shape of the particles and the size [135,136].

### 5.3. Electron Microscopy-SEM (Scanning Electron Microscopy)

Scanning electron microscopy (SEM) together with Energy Dispersive X-ray Analysis (EDSX) is a technique which offers information related to the morphology of nanoparticles of the sample, offers dimensional measurements on the component particles and a qualitative microanalysis through the element identification, as well.

In the case of nanoparticles, structural information is needed, as well, therefore electron microscopy is a necessary tool. Based on a high energy electron beam under vacuum, the sample is bombarded and information about inside morphology can be identified. EDSX is an electronic module attached to the SEM equipment. It is highly recommended for an elemental analysis since SEM penetrates the material internal structure, produces ionization of the atoms by electrons from the deeper electron levels. In the case of silver nanoparticles, SEM images can prove the spherical form desired, while EDSX can show that nanoparticles are actually made of silver. SEM images can determine possible agglomeration of nanoparticles, as well, therefore SEM images could complete the results of DLS size analysis.

**Figure 10:**
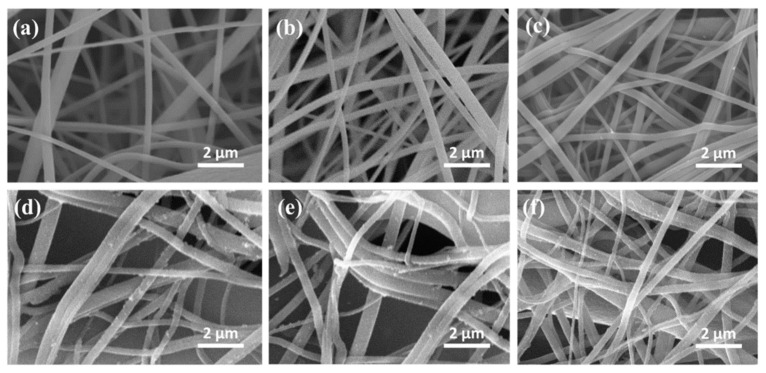
SEM analysis for: (**a**) cellulose acetate, (**b**) cellulose nanofibers, (**c**) orange-oil cellulose nanofibers, (**d**) Ag-cellulose nanofibers, (**e**) Ag-orange oil 5% cellulose nanofibers, (**f**) Ag-orange oil 10% cellulose nanofibers. Reprinted from an open source article [137].

SEM images are essential for silver nanoparticles characterization in all types of applications. Their shape is observed while the sizes can also be confirmed after DLS measurements are obtained. In most of the studied particles are investigated individually or after they are integrated in a matrix. In a recent study, the antibacterial effect of silver nanoparticles was tested using cellulose membrane as support. Comparing the silver nanoparticles scaffolds with orange essential oil-based scaffolds and with coated-silver nanoparticles scaffolds, the porosity and the release properties of cellulose scaffolds were investigated through SEM, as it can be observed in Figure 10 [137], published under open access.

The results of the study revealed that Ag NPs integration in a hydrogel matrix can modify the size of cellulose nanofibers and the final properties of the material, starting from approximately 400 nm (Figure 10a,b). In the case of orange oil there were no visible transformations of this kind. The dimensions were maintained around 400 nm while the oil evaporated in the vacuum chamber. In case of Ag NPs, it was proved an increase in nanofibers dimension from 400 to approximately 430 nm, therefore it can be concluded that the spherical nanoparticles were bound to the fibrous structure. Beside the other analysis performed, these SEM images proved that encapsulation of Ag NPs in an orange oil shell will not reduce their antibacterial activity and their ability to be trapped in polymeric matrix pores for release applications [137].

**Figure 11:**
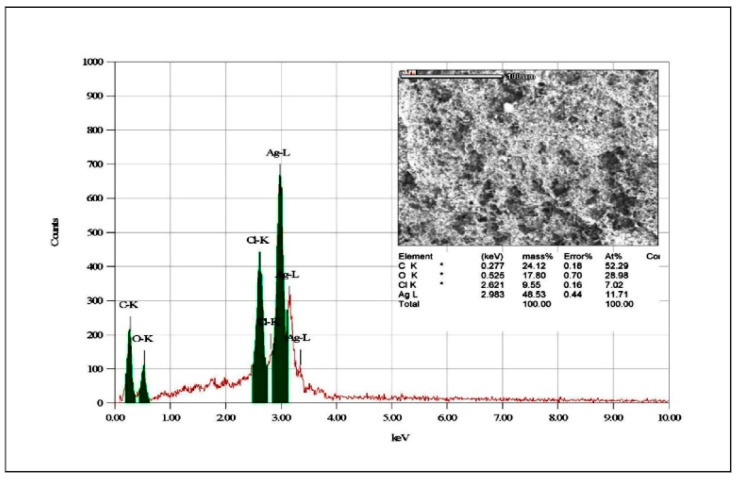
EDS spectrum for elemental analysis in Ag-NPs synthetized through *Urtica diocia* Leaves. Retrieved from an open source article [138].

Binsalah et al. [138] proved using the EDS module attached to SEM equipment that Ag NPs obtained through green chemistry exhibits Ag in the highest quantity in a pure form (Figure 11). Even if the elemental analysis showed variety in elements, the high peak associated with Ag confirms that this metal can perform its antibacterial activity even beside other reactive elements. The SEM and TEM images exposed a spherical form for nanoparticles which did not showed aggregations, so a conclusion can be that they will not be accumulated in the tissues in large aggregates. The average size was under 20 nm, so they have a classical morphology for an antimicrobial character. Using the classical characterization method, the nanoparticles proved to have antibacterial properties against Gram-negative and Gram-positive bacteria [138].

### 5.4. Nuclear Magnetic Resonance (NMR)

Nuclear magnetic resonance is a spectroscopic technique able to offer information regarding structural properties. Based on the magnetic resonance phenomenon, the method described since 1940 can determine molecular interactions between molecules and different chemical species. The physical principle of this technique is based on radiofrequency radiation with the atomic nuclei in the sample. The interactions involve an exchange of energy in the sample and a modification of the atomic nuclei motion. [139,140].

All the parameters from this technique are controlled by the electronic system. The data acquisition is also integrated in this system, enabling an NMR to be obtained at the end of the process. The spectrum obtained is correlated to the peaks obtained from different intensities depending on the magnitude named in literature „chemical shift”. This shift is obtained from the Larmor frequency of various nuclei found in the sample [141]. The spectrum is a proportionality between the intensity of the signal and the ^1^H chemical shift of each atomic nuclei type from the sample. In the case that the sample studied is composed of similar chemical groups, the spectrum will show a single peak for one of the groups, thus enabling the determination of the type of atoms. NMR spectrum offers information on the molecules by identifying the chemical groups. It is a very sensible method used for internal information of the protons of the samples.

Uznanski et al. [142] performed a comparative study between amine and silver carboxylate chemical substances in order to be used for Ag NPs synthesis. The study was based on the chemical bonds analysis formed between silver ions and the two substances. Ag NPs can be synthetized through an innovative chemical synthesis based on aliphatic amines and silver carboxylates. In such reaction, the nanoparticles are entrapped in the carboxylate group, while the tertiary amines can create the reducing medium necessary for the reaction. Using thermal decomposition, the silver nanoparticles can be produced also at lower temperatures. The authors used ^1^H and ^13^C NMR spectroscopy to observe the complexes amine/carboxylate and to understand better the chemistry of stale Ag NPs. formed. The study showed the amine, in this case, does not act as a reducing agent, but it helps the decomposition process. Once the reaction is complete, amine and carboxylates interact, while the Ag NPs is obtained. The others characterization methods proved an average size of about 5 nm for Ag NPs, so the method and the results obtained from NMR spectroscopy were useful for this innovative method which will be further investigated [142].

## 6. Conclusions

Silver nanoparticles are metal-based materials intensively studied and integrated in various forms of biomaterials. Since their dimensions are so small, their integration in hydrogels or devices for soft and hard tissues regeneration seems to be an effective alternative to existent expensive treatments. Their versatility, optical activity, adaptability to chemical changes at their surface, their possibility of functionalization and their well-known antibacterial effect encouraged interesting studies over time. Ag NPs shape, size or surface have proved to be possible in various models since the synthesis methods are so varied.

Over time, many types of synthesis were tested for Ag NPs depending on the final application. The chemical or physical methods described through this paper showed that Ag NPs are suitable for simple or complex equipment. Green chemistry, the newest approach in which natural compounds are used as reducing agents, can create stable system with strong antibacterial effect comparable to classical methods. Systems based on Ag NPs are used nowadays for antibacterial effect, targeted therapies or drug release. Their size, an important aspect, can be determined using techniques such as DLS, SEM or AFM. Determination of size distribution is an essential step since their effect is in good correlation with their size. It remains a clear affirmation that silver nanoparticles exhibit powerful antibacterial effects while they remain in the nano domain, therefore deeper investigations on their mechanisms are required for medical applications.

## Figures and Tables

**Table 1:** Main synthesis methods for silver nanoparticles.

Category	Type of Method	References
**Physical methods**	Laser ablation	[73]
	Ball milling	[74]
	Ion sputtering	[75]
**Chemical methods**	Chemical reduction	[76]
	Micro-emulsion	[77]
	Electrochemical cell	[78]
	Hydrothermal method	[79]
**Green synthesis**	Based on plants/bacteria/fungi	[80]

**Table 2:** Chemical reduction method for silver nanoparticles synthesis using various chemical agents.

Silver Source	Reducing Agents	Stabilizing Agents	Form of Ag NPs	References
**Silver nitrate/Silver citrate/Silver acetate**	Sodium borohydride	Sodium borohydride	SPHERES	[76]
Silver nitrate	Hydrazine	Polyvinyl pyrrolidone (PVP)	TRIANGULAR	[82]
Silver nitrate	Hydrazine Ascorbic acid	Polyvinyl alcohol (PVA)	SPHERES/CUBES	[83]
**Silver nitrate/Silver citrate**	Potassium bitartrate Dimethylformamide (DMF)	Polyvinyl pyrrolidone (PVP)	SPHERES/CUBES/BARS/WIRES	[84]
**Silver salts**	Dimethylformamide (DMF)	Polyvinyl pyrrolidone (PVP) Aminopropyltriethoxysilane (APS)	SPHERES (surrounded by a SiO_2_ layer)/PRISMS	[84]
**Silver nitrate**	Ethylene glycol	Polyvinyl pyrrolidone (PVP)	BARS/WIRES	[85]
Silver nitrate	Ascorbic acid	CTAB + NaOH	RODS/WIRES	[86]
Silver nitrate	Polyethylene glycol (PEG)	Polyvinyl pyrrolidone (PVP)	WIRES	[87]
**Silver nitrate**	D-Glucose	Starch	SPHERES/CUBS	[84]

**Table 3:** Green synthesis of silver spherical nanoparticles using, as a silver precursor, AgNO_3_.

	Name	Size (nm)	References
**Plants**			
	*Calotropis procera*	**4–25**	[108]
	*Olax scandens*	**10–110**	[109]
	*Datura metel*	**12**	[110]
**Bacteria**			
	*Bacillus cereus*	**20–40**	[111]
	*Bacillus cecembensis*	**3–18**	[112]
	*Bacillus methylotrophicus*	**10–30**	[113]
	*Brevibacterium frigoritolerans*	**10–150**	[114]
	*Actinobacteria*	**5–80**	[115]
**Fungi**			
	*Schizophyllum commune*	**54–99**	[116]
	*Trichoderma viride*	**5–40**	[117]
	*Endophytic fungus*	**25–30**	[118]
	*Penicillium fellutanum*	**5–25**	[119]

**Table 4:** Advanced characterization techniques for Ag-NPs investigation.

Characterization Method	Property Studied
**Scanning Electron Microscopy**	Size determinations. Shape. Aggregation
**Transmission Electron Microscopy**	Size determinations. Shape. Aggregation
**Atomic Force Microscopy**	Size determinations. Shape. Structure
**Dynamic Light Scattering**	Average size determinations
**X-ray analysis**	Structure. Crystallinity
**Nuclear Magnetic Resonance**	Structure. Composition
**Mass spectrometry**	Composition. Structure. Molecular weight
**UV-VIS spectroscopy**	Size determinations. Release properties
**Fourier-transform infrared spectroscopy**	Functionalization
**Gene expression**	Toxicity on cells. Stress gene expression
**Reactive Oxygen Species detection**	Toxicity on cells
**Agar well diffusion model**	Zone of inhibition due to bactericidal activity
